# Why Do We Agree to Disagree? Agreement and Reasons for Disagreement in Judgements of Intentional Self-Harm from Coroners and a Suicide Register in Queensland, Australia, from 2001 to 2015

**DOI:** 10.3390/ijerph21010052

**Published:** 2023-12-30

**Authors:** Stuart Leske, Bridget Weir, Ghazala Adam, Kairi Kõlves

**Affiliations:** 1 Australian Institute for Suicide Research and Prevention, World Health Organization Collaborating Centre for Research and Training in Suicide Prevention, School of Applied Psychology, Griffith University, Brisbane, QLD 4122, Australia; s.leske@griffith.edu.au (S.L.); bridget.weir@qut.edu.au (B.W.); ghazadam20@gmail.com (G.A.); 2 UQ Poche Centre for Indigenous Health, The University of Queensland, Brisbane, QLD 4066, Australia

**Keywords:** coroners, coronial, mortality, intentional self-harm, National Coronial Information System, NCIS, suicide

## Abstract

Suicides are likely to be underreported. In Australia, the National Coronial Information System (NCIS) provides information about suicide deaths reported to coroners. The NCIS represents the findings on the intent of the deceased as determined by coroners. We used the Queensland Suicide Register (QSR) to assess the direction, magnitude, and predictors of any differences in the reporting of suicide in Queensland. Therefore, we conducted a consecutive case series study to assess agreement and variation between linked data from the NCIS and QSR determinations of suicide for all suicide deaths (*N* = 9520) in the QSR from 2001 to 2015 recorded from routinely collected coronial data. The rate of concordance between the QSR and NCIS for cases of intentional self-harm was 92.7%. There was disagreement between the findings in the data, since 6.3% (*n* = 597) were considered as intentional self-harm in the QSR but not in the NCIS, and, less commonly, 0.9% (*n* = 87) were considered intentional self-harm in the NCIS but not in the QSR. Overall, the QSR reported 510 more suicides than the NCIS in 15 years. These findings indicate that using suicide mortality data from suicide registers may not underreport suicide as often.

## 1. Introduction

Accurate suicide mortality data help to plan and evaluate public health policy and interventions [1,2,3], inform resource allocation for suicide prevention, serve as a marker of community health and wellbeing [4], and assist with estimating the economic cost of suicide and addressing the stigma surrounding suicide [1,2,3,5]. Suicide underreporting occurs internationally, and in some regions, this undermines usable suicide mortality data. Moreover, reliable and valid suicide data may be rarely available in developing countries. In countries with coronial systems, research has shown that coroners may differ in findings of suicide, the interpretation of evidence, and the interpretation and application of coronial law relating to suicide determination in practice [4,6,7]. Undercounting or misclassification of suicide is of great concern within the academic and public health sphere [8,9]. 

Internationally, the accuracy and reliability of officially reported suicide statistics has been widely researched [10,11]. A 2012 systematic review [11] of the reliability of suicide statistics supports findings of a general underreporting of suicide internationally, though it indicated that further studies of suicide statistics were still needed. Officially recorded mortality statistics may underestimate suicides due to the differential classification of death as due to other causes. Commonly, deaths which on their face appear to be suicides but do not involve a clear intent to end one’s life may be determined to be unintentional, accidental, or undetermined in nature.

In Australia, there have been several reports and papers dedicated to the accuracy of suicide mortality statistics [1,2,3,5,9,12,13], with most [1,2,3,5,9,12] about the Australian Bureau of Statistics’ (ABS) public reporting of this data. We refer readers to the most recent of these papers [12] to understand that historical context. The ABS’s use of the NCIS is primarily to obtain additional information about external-cause deaths that they have received initial information about via Births, Deaths and Marriages Registries. The NCIS is an online repository containing information on all deaths reported to a coroner, including suicide deaths, since 2000. Data for all Australian states and territories (except Queensland) are available from 1 July 2000. Queensland data are available from 1 January 2001 and New Zealand data from 1 July 2007. Historically, the ABS reported the figures of the NCIS; however, after concerns were raised about this process [14], the ABS revised their processes to enable additional scrutiny by ABS staff of the probability of a death being a suicide.

In the context of suicide mortality, the NCIS has been used for key, informative, nationwide studies in Australia, such as the suicides of different occupational groups [15,16,17,18,19,20,21] and youth [22]. However, researchers acknowledge that the NCIS may underreport the occurrence of suicide [3,13,16], due to a lack of standardization between coroners and across jurisdictions [7,23].

Previous studies of the NCIS have found that there were discrepancies between the ABS and the NCIS concerning the International Classification of Diseases Tenth Revision (ICD-10) cause of death codes [24], ICD-10 cause of death codes and the intent type in the NCIS measure of intentional self-harm (ISH) [25], and between ISH in the NCIS and a state forensic services department [13]. The NCIS has responded with commentaries [26,27] on some of these issues. Outside Australia, one study [28] of suicides in England in 2005 has assessed how coroners’ verdicts diverged from researchers’ verdicts of whether a death was a suicide or not. Since that study occurred, England has switched from the criminal standard of proof (‘beyond reasonable doubt’) to using the civil balance of probabilities method to determine suicide deaths at inquest [29], which more closely aligns with the research criteria of defining a suicide death.

Following on from these studies, our objective was to assess agreement between the NCIS and codes from an independent suicide mortality register for all suspected suicides reported to that register from 2001 to 2015. Such a study would benefit the NCIS and NCIS users by helping to understand the direction, magnitude, and explanations for differences between various data collections in terms of the overall numbers of suicides.

## 2. Materials and Methods

### 2.1. Study Design and Data Sources

This is a consecutive case series study of all suspected suicides reported to the Queensland Suicide Register (QSR). These deaths are all cases that the police consider as suspected suicides that occurred in Queensland, Australia, irrespective of final determination. Australia has six states and two territories, and of these, Queensland has the third-largest population after New South Wales (NSW) and Victoria. ABS figures [30] show that in the last ten years, Queensland has recorded the second-highest number of suicides of all jurisdictions in Australia, after NSW, in all years except one. These figures also show that for the last ten years, Queensland has an age-standardized suicide rate that is above the Australian average and ranges from being the second-highest- to the fourth-highest rate of all jurisdictions. We used QSR data from 2001 to 2015, as Queensland data in the NCIS are available from 1 January 2001 onwards, and 2015 was the last year complete data were available in the QSR at the time the data were extracted for analysis on 11 November 2021. There were no further eligibility requirements other than the year of death to be included for analysis.

Our outcome variable was whether a death was determined to be a suicide or not in NCIS data. These deaths are coded in the NCIS as deaths due to external causes. Within deaths due to external causes, information on intent refers to the intent determined by coroners once they have finalized their investigation into the death. Possible intent types include ‘unintentional’, ‘intentional self-harm’ (ISH), ‘assault’, ‘undetermined intent’, or ‘unlikely to be known’. NCIS case data are coded by court staff in each jurisdiction based on the coronial file, and that intent is coded according to the findings of the investigating coroner. In the NCIS, ISH is defined as any ISH resulting in death, so it does not differentiate between instances where the deceased intended to die because of their actions and instances where the deceased intended to self-harm without intending to die. It is therefore not possible to conclusively state that all ISH deaths are suicides. Alongside deaths due to natural or external cause(s) in this field, NCIS also has another code for ‘Body not recovered’. Under the NCIS’s field ‘Case type – Completion’, we also matched those held in the QSR with those assigned as experiencing ‘Death due to natural cause(s)’ in the NCIS. Further information on NCIS classifications is available in the NCIS system manuals (coding manual and data dictionary), both available online [31]. For simplification, the NCIS variable was coded as ‘0’ for most categories and ‘1’ for ISH. 

The standard procedures for the QSR have a probabilistic classification of each death, assigning the probability of a death being a suicide as ‘unlikely’, ‘possible’, ‘probable’, or ‘beyond reasonable doubt’ (later changed to ‘confirmed’). Consistent with all QSR analyses, only deaths considered as ‘probable’ or ‘confirmed’ were considered suicides. Given that the NCIS assesses a wider range of reportable deaths, there is no equivalent probabilistic classification approach, and therefore no equivalent code to ‘probable’, in the NCIS. Possible suicides could also be an accident, illness, or homicide, which may account for differences between the two datasets. Suicides are confirmed if the person communicates their intent to die by suicide, verbally or in writing, in relation to the suicide attempt that leads to their death. The decision-tree guiding this process and the flow of information into the QSR from the NCIS and other data sources from the most recent annual report of this system are available online [32].

### 2.2. Statistical Analysis

We conducted a binary logistic regression to understand what variables predicted discordance while accounting for other variables that may predict this outcome. This analysis would provide information on the primary variables responsible for discordance that would be useful to those seeking to reconcile differing judgements of suicide between coroners and other death investigators. For the binary logistic regression models, the outcome variable was coded as ‘0’ for agreement between the NCIS and the QSR (intentional self-harm in the NCIS and a ‘probable’ or ‘beyond reasonable doubt’/‘confirmed’ suicide in the QSR) and ‘1’ for a determination of suicide by QSR staff and not ISH in the NCIS. As this was a data-driven decision, we elaborate in the results on the reasons why we did not choose a multinomial model that also had an outcome of ISH in the NCIS and not a suicide in the QSR. In the binary logistic regression model, we considered all other variables as predictors, since they were all determined before the death by suicide. We were not interested in a specific exposure, did not have potential confounders of this exposure–death relationship, and we did not study any effect modifiers. Predictor variables of interest included age; year of death; sex; Indigenous status; marital status; employment status; country of birth, remoteness area of the residential address; lifetime or 12-month (two variables) communication of intent to die by suicide; lifetime or 12-month (two variables) suicide attempts; suicide note left; whether alcohol was consumed before suicide; a police- or coroner-reported diagnosis of depression, bipolar disorder, anxiety, substance use disorder or neurocognitive disorder; general practitioner, inpatient, outpatient or other service treatment for a psychiatric condition (4 variables); living arrangements; and the life events of relationship problems, sexual abuse, interpersonal conflicts, or recent or pending unemployment (4 variables).

In terms of data sources and their measurement, we derived most information from the police report of death to a coroner [33], received directly from Queensland Police Service or Coroners Court of Queensland staff, or a coroner’s findings and notice of completion of coronial investigation [34]. To reduce the missingness of several variables, data are also extracted from the NCIS on Indigenous origin (as coded by NCIS coders), country of birth, and Indigenous status derived from state-based registries of births, deaths and marriages, occupation, employment status, and marital status.

We attempted to minimize study bias in several ways. We tried to minimize detection- and misclassification bias in the QSR by using a structured decision-tree and having one person enter and another person check each judgement made on whether a death was a suicide or not. We sought to minimize recall bias by relying on police reports completed soon after a death had occurred. We tried to minimize selection bias by scrutinizing additional potential deaths by suicide in the NCIS to ensure the QSR captured the population of people dying by suicide in Queensland.

The study size was determined by whatever data we had complete information on in all variables included in the multivariable analysis. For this analysis, we collapsed variables to the least degree possible when the models run either did not provide an estimate for the category or provided an estimate that was implausible.

We compared inter-rater agreement with several measures, including percent agreement, Brennan and Prediger [35], Cohen [36,37]/Conger’s [38] Kappa, Scott [39]/Fleiss’ Pi [40], Gwet’s AC [41,42], and Krippendorff’s Alpha [43,44,45]. Although we reported all these as they were produced as part of the output, we preferred Krippendorff’s Alpha in the event of disagreement due to its ability to accommodate multiple raters.

For predicting the outcome variable, we employed a binary logistic regression model. To examine the functional form of the continuous variables age and incident year, we employed multivariable fractional polynomials and restricted cubic splines with 5 knots. We also included these variables, categorized to the least degree possible, in binary logistic regressions in the Supplementary Materials.

We conducted all analyses in Stata, version 13.1 [46]. We used several user-written commands: MDESC [47] to tabulate the prevalence of missing values, COLLIN [48] to examine variance inflation values during multicollinearity checks, BACON [49] to identify multivariate outliers using the blocked adaptive computationally efficient outlier nominators (BACON) algorithm [50], and KAPPAETC [51] to evaluate inter-rater agreement. Tables were created using asdoc, a Stata program written by Shah [52].

### 2.3. Ethics

The QSR project has ethical approval from the primary body, the Victorian Department of Justice and Community Safety’s Human Research Ethics Committee (JHREC CF/18/12771) and Griffith University’s HREC (2010/537) as the secondary review body.

All investigators had full access to the database population used to create the study population. Data cleaning methods used in the study included locating original source files to clean missing data and using information from other data sources to reduce the missingness on variables included in the models. The study involved person-level data linkage between data received from the Coroners Court of Queensland (CCQ) and the NCIS. In terms of the linkage, from 2001 to 2015, 0.84% of cases received from the CCQ could not be located in the NCIS.

### 2.4. Reporting Guidelines

As this was an observational study of routinely collected data, we report (Supplementary Table S1) per the Strengthening the STrengthening the Reporting of OBservational studies in Epidemiology (STROBE) Statement [53] and the REporting of studies Conducted using Observational Routinely-collected Data (RECORD) Statement [54].

## 3. Results

In total, there were 9520 suspected suicides from 2001 to 2015 available for analysis. All suspected suicides had a determination made by QSR staff, while we were able to import determinations for 9427 (99%) suspected suicides from the NCIS. Table 1 shows the determinations in the QSR and the coroners’ determinations in the NCIS, with their column proportions as percentages. 

Table 2 shows measures of inter-rater agreement, including all measures for comparison. While some measures like Gwet’s Agreement Coefficient (AC) have excellent agreement, three, including our preferred measure, have similarly fair agreement.

Supplementary Tables S2–S4 show the numbers and proportions of each code for each variable included as a predictor in the binary logistic regression. Missing data on predictor variables excluded 4.45% of people (*n* = 424) from the analysis. We checked whether the outcome predicted missingness in a logistic regression, and it did not (odds ratio (OR) = 0.69, 95% CI = 0.37 to 1.26, *p* = 0.2), so we proceeded without analyzing multiply imputed data since the proportion of missingness was under 5% and unrelated to the outcome.

**Figure 1:**
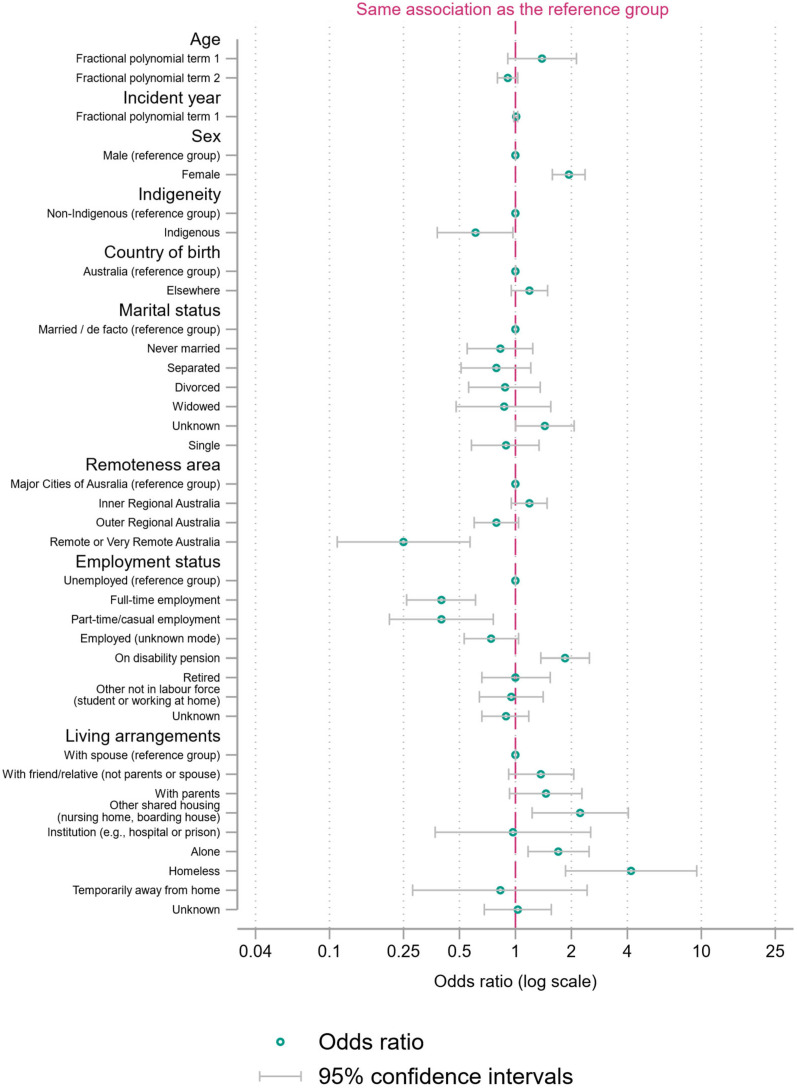
Binary logistic regression with fractional polynomial terms for age and incident year (ORs with 95% CI), demographic variables in the regression.

**Figure 2:**
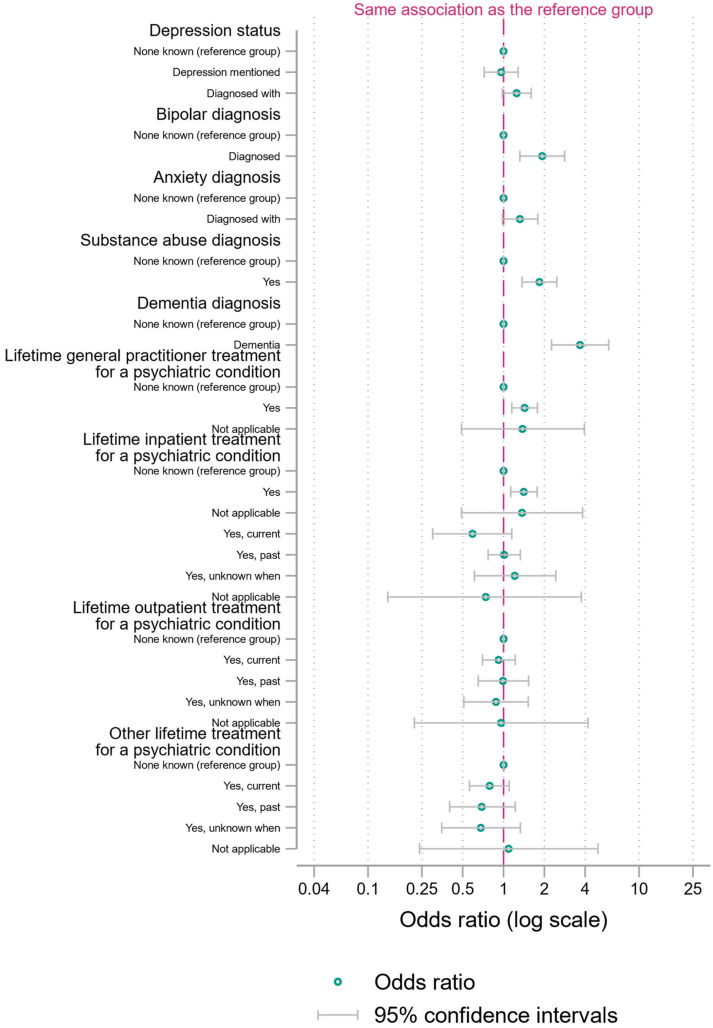
Binary logistic regression with fractional polynomial terms for age and incident year (ORs with 95% CI), psychiatric variables in the regression.

**Figure 3:**
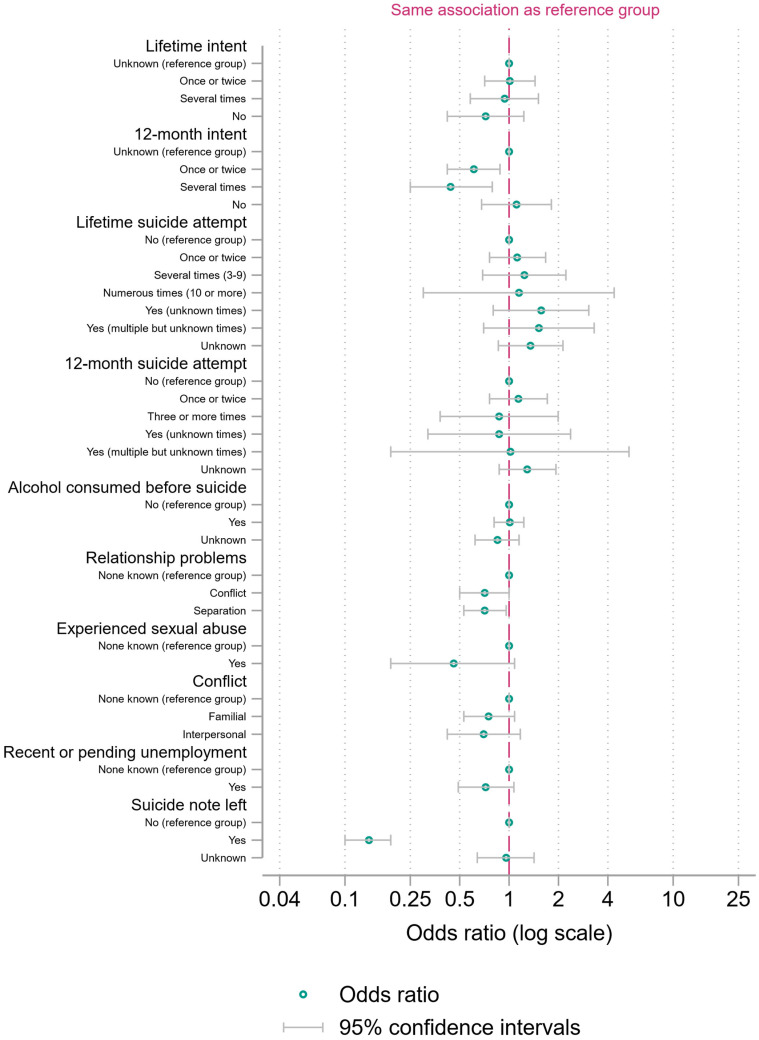
Binary logistic regression with fractional polynomial terms for age and incident year (ORs with 95% CI), suicide-related and life event variables in the regression.

We present a main binary logistic regression model with fractional polynomial terms for age and incident year (Figure 1, Figure 2 and Figure 3 and Supplementary Table S5). We present two sensitivity analyses (Supplementary Tables S6 and S7): a binary logistic regression with restricted cubic splines as an alternative approach to modelling the functional form of continuous variables and a model with the two continuous variables categorized into 10-year age groups or five-year periods of incident years.

The lowest *p*-value for any age term was 0.1, for one of the fractional polynomial terms. The findings for incident year differed depending on how it was treated, with little association in the fractional polynomial and restricted cubic spline approaches. However, the odds ratio for discordant verdicts for an incident year between 2006 and 2010 was 1.3 times higher (95% CI = 1.02 to 1.68, *p* = 0.04) compared to the years 2001 to 2005. Estimates for other variables changed little across the three models.

There were several elevated odds ratios. Demographically, the odds of discordant judgements were 1.94 times higher (95% CI = 1.58 to 2.36) when the person dying by suicide was female. There were odds of discordance 1.44 times higher (95% CI = 1.00 to 2.07) for those with unknown marital status, compared to those with married or de facto status.

In terms of employment status, the odds of discordant judgements were 0.4 times as high for those in full-time employment (95% CI = 0.26 to 0.61) and part-time or casual employment (95% CI = 0.21 to 0.76) compared to the base category of unemployment. Inverted, this meant that the odds of discordant judgements were 2.5 times higher when a person was unemployed, compared to the person being in full-time employment (95% CI = 1.65 to 3.78) or part-time or casual employment (95% CI = 1.32 to 4.7). The odds of suicide in the QSR vs. not a suicide in the NCIS were also 1.85 times higher for suicide decedents on a disability pension (95% CI = 1.37 to 2.50) than those unemployed. Those employed in an unknown mode had odds of discordant judgements 0.74 lower (95% CI = 0.53 to 1.04, *p* = 0.08), which, when inverted, meant that people recorded as unemployed had odds of discordant judgements 1.35 times higher (95% CI = 0.96 to 1.89). Lastly, compared to the unemployed, the odds of discordant judgements were quite similar for people reportedly retired (OR = 1.00, 95% CI = 0.65 to 1.54) or not in the labor force (OR = 0.95, 95% CI = 0.64 to 1.41) and, to a lesser extent, those with unknown employment status (OR = 0.89, 95% CI = 0.66 to 1.18).

Considering the remoteness area of the residential addresses of decedents, the odds of discordance were 0.25 (95% CI = 0.11 to 0.58) for people in remote areas, relative to those in major cities. Inverting the OR, this meant that the odds of differing judgements were 4.05 times higher (95% CI = to 1.74 to 9.5) in major cities compared to remote and very remote areas. The odds of discordant judgements were also 0.79 times lower in outer regional areas (95% CI = 0.6 to 1.04, *p* = 0.09), or, inverted, 1.27 times higher (95% CI = 0.96 to 1.67) in major cities than outer regional areas. Lastly, the odds of discordant judgements were 1.19 times higher (95% CI = 0.95 to 1.48) in the suicides of people residing in inner regional areas, relative to those in major cities.

There were varying odds ratios by living arrangements. Specifically, the odds of discordance for a homeless person were 4.2 times higher than if the person lived with a spouse (95% CI = 1.86 to 9.45). The odds of discordant judgements were 2.23 times higher (95% CI = 1.23 to 4.05) for people who lived in other shared housing, compared to those living with their spouse. The odds of discordance were also 1.70 times higher (95% CI = 1.17 to 2.49) when the person lived alone, compared to people living with their spouse. People living with their parents also had odds of a discordant judgment 1.46 times higher (95% CI = 0.92 to 2.06). The OR for people who lived with friends/relatives was also elevated, at 1.37 (95% CI = 0.92 to 2.06). 

In terms of mental health conditions, the odds of discordant judgements were 3.67 times higher (95% CI = 2.26 to 6.00) when the deceased had dementia. The odds of discordant judgements were also 1.93 times higher (95% CI = 1.32 to 2.82) when the deceased had been diagnosed as bipolar. Of a similar magnitude, the odds of discordant judgements were 1.84 times higher (95% CI = 1.37 to 2.46) when the deceased had a substance abuse condition.

The last three categories had ORs more compatible with null hypotheses of no difference than not: living in an institution (OR = 0.97, 95% CI = 0.94 to 0.37), living temporarily away from home (OR = 0.83, 95% CI = 0.28 to 2.43), and unknown living status (OR = 1.03, 95% CI = 0.68 to 1.56).

The odds of discordance were lower when the deceased had communicated their intent to die by suicide in the past 12 months: 0.61 times lower (95% CI = 0.42 to 0.88) when the deceased had communicated intent once or twice in the last 12 months and 0.44 times lower (95% CI = 0.25 to 0.79) when the deceased had communicated intent to die by suicide several times, both versus unknown times. Inverting both odds ratios for easier interpretation, the odds of discordance for people with unknown communication of intent in the past 12 months were 1.64 times greater (95% CI = 1.13 to 2.36) vs. intent communicated once or twice and 2.26 times greater (95% CI = 1.27 to 4.02) vs. intent communicated several times. Lastly, data were more compatible with the null hypothesis of no difference for no communication of intent vs. unknown communication, with a similar odds ratio, an OR of 1.11 (95% CI = 0.68 to 1.81). The same findings were not observed regarding the deceased communicating their intent to die by suicide in their lifetime. There were not lower odds of discordance if the deceased had communicated intent once or twice (OR = 1.01, 95% CI = 0.71 to 1.44) or several times (OR = 0.93, 95% CI = 0.58 to 1.51), compared to the referent category of unknown. However, the OR was lower, and the *p* value much lower, for the no category (OR = 0.72, 95% CI = 0.42 to 1.23), indicating more discordance when there was unknown intent vs. no intent communicated in the decedent’s lifetime.

The odds of discordance were 1.41 times higher (95% CI = 1.13 to 1.77) when the deceased had received treatment from a GP for a psychiatric condition in their lifetime, relative to the odds for the no category. Although there was a similar OR (1.37) for the ‘not applicable group’ (those without a diagnosed psychiatric condition), the smaller numbers here made the confidence intervals much wider (0.49 to 3.83). 

For Indigenous status, the odds of discordance were 0.6 times lower when the deceased was Indigenous (95% CI = 0.38 to 0.97). Inverted, the odds of discordance were 1.67 times higher (95% CI = 1.03 to 2.65) when the deceased was non-Indigenous, compared to Indigenous.

In terms of relationship problems, there were lower odds of discordance when the deceased had experienced relationship conflict (OR = 0.71, 95% CI = 0.50 to 1.00) or separation (OR = 0.71, 95% CI = 0.53 to 0.96). Inverted, the odds of discordance for people experiencing neither relationship conflict nor separation were 1.41 times higher (95% CI = 1.00 to 1.98) than for those experiencing conflict and 1.40 times higher than for those experiencing separation (95% CI = 1.04 to 1.88). 

In terms of sexual abuse, the odds of discordance were 0.46 times lower (95% CI = 0.19 to 1.08) when the deceased had experienced sexual abuse. This meant that the odds of discordance were 2.19 times higher (95% CI = 0.92 to 5.21) when the deceased had no known experience of sexual abuse.

The odds of a discordant judgement were 0.14 times (95% CI = 0.10 to 0.19) as high (or the odds of concordant judgements 7.2 times higher) when decedents left a suicide note, compared to no suicide note being left.

## 4. Discussion

The objective of this study was to assess the degree of concordance between verdicts of ISH and other causes of death by coroners, reflected in the NCIS, and researcher-based decisions of suicide from a state-based suicide register operating independently of state and federal government agencies. We also sought to understand predictors of the most common discrepant verdicts. 

While percent agreement was high, measures of inter-rater reliability were low. This is expected since the two groups of raters have different standards—one has more onerous legal criteria, while the other has broader health research criteria. However, the absolute number of discrepancies over a 15-year period is more than desirable. Our findings suggest that coroners’ judgements of ISH have low inter-rater reliability concerning judgements of suicide in the state of Queensland.

The multivariable modelling process indicated that discrepant judgements were not confined to any one group, as several factors in different domains predicted discrepant judgements. In addition, some of the reduced odds were unsurprising (for those who left a suicide note or communicated their intent to die by suicide). 

Beyond these findings, however, there appears to be higher odds of discrepant judgements when suicides involve those who die by suicide at lower rates (e.g., females) or in more complex circumstances (e.g., those with dementia). Notably, some traditional risk factors for suicide were associated with reduced odds of discordance (relationship conflict or separation, sexual abuse), but others were instead associated with higher odds of discordance (a diagnosis of bipolar disorder or a substance abuse condition).

In terms of comparing our findings with those of previous studies, our percent agreement of 93% is higher than the 65.4% recorded between researchers and coronial verdicts in 593 deaths reviewed in England in 2005 [28] and higher than the 69% recorded between coroners’ verdicts and the National Office for Suicide Prevention (NOSP) for deaths in Ireland between 2015 and 2018 [60]. More similarly, an examination of potential misclassification of 998 army suicides led to 8.2% of the non-suicide cases being considered a possible, probable, or definite suicide [61].

Similarly, and expectedly, a suicide note emerged as a predictor of coronial judgements of suicide, or lower odds of discordance, in Palmer et al.’s study [28] and ours. However, we did not have the same findings for age or marital status that they observed. In Australia, our findings on sex correspond to those in a South Australian study [13], which reported higher numbers of suicides by women than coroners did, as recorded in the NCIS. In Ireland, there was agreement between coroners’ verdicts and the NOSP for 71% of the probable suicide deaths by men and 64% for women [60].

Some caveats should be noted when comparing our results to other studies. The findings of Palmer et al. [28] are not directly comparable to ours, as they constructed logistic regression models to examine the factors predicting coroners’ determinations of suicides rather than other deaths and not the concordance or otherwise of these judgements with health research criteria. Likewise, the Irish Probable Suicide Deaths Study of deaths from 2015 to 2018 examined descriptively, and with chi-square bivariate tests (but not odds ratios), associations between coroners’ verdicts and those of the Irish Health Services Executive National Office for Suicide Prevention. 

There are several limitations to this study that readers should consider as they interpret the findings. These findings do not generalize to any other jurisdictions in Australia as this study has looked only at Queensland, and the existence of a suicide register outside the state government exists only in Queensland. On the NCIS side, there may be other individual or regional factors that affect how coroners determine ISH in other jurisdictions. There are no objective criteria for what constitutes ISH or a death by suicide, so independent judgements made by staff of a suicide register are not necessarily the gold standard but rather a system that is not constrained by legal processes and precedents. There is the potential for misclassification bias among both sets of coders. We used data collected over a 15-year period, which would have witnessed many changes in the practices of coroners determining ISH or the QSR determining suicides. Both sets of coders rely on forms completed by reporting police officers at the scene, with neither coder attending the scene. These details collected by police may be subject to change by the time the case is closed by the coroner. The Coroners Court of Queensland can access more material when making judgements about suicides, especially when inquests into the deaths were held. The crude nature of the data being collected, with much of it being nominal variables, means that there may be more complex variables that are more predictive of the outcome and that there may be misclassification bias beginning when data are first collected about the suicide (e.g., misclassification of Indigenous people as non-Indigenous). Still, we had only a small proportion of missing data for all variables, although we treated unknown categories as data since these options to report some factor as unknown exist in the police report.

Overall, this study provides further evidence that there may be some underreporting of ISH in the coronial system as reflected in the NCIS. This is a well-known limitation of data on sudden and unexpected deaths and, as mentioned earlier, acknowledged as a limitation by users conducting analyses of suicide mortality data held by the NCIS.

## 5. Conclusions

Our findings, to our knowledge, represent the largest study thus far examining the concordance between coroners’ judgements of intentional self-harm and a suicide register. After accounting for chance agreement, we found poor agreement between the two data sources, likely reflecting the different criteria (legal and health research) used in the different processes. Some predictor variables were unsurprising, while others provide researchers and others accessing the NCIS with useful information about the characteristics of deaths that might be suicides according to other coding frameworks.

## Figures and Tables

**Table 1:** Concordance between intentional self-harm in the National Coronial Information System with suicides in the Queensland Suicide Register.

	Intentional Self-Harm in the National Coronial Information System					
Queensland Suicide Register Dichotomous Variable for Suicide	Yes—*n*	Yes—%	No—*n*	No—%	Total—*n*	Total—%
Not suicide	87	1.0	439	42.4	526	5.6
Suicide	8304	99.0	597	57.6	8901	94.4

**Table 2:** Measures of inter-rater agreement for National Coronial Information System and Queensland Suicide Register judgements, 2001 to 2015 (*N* = 9427).

Measure of Agreement	Coefficient	SE	T-Value	*p*-Value	95% CI LL	95% CI UL	Level of Agreement
Percent Agreement	0.93	<0.01	327.3	<0.001	0.92	0.93	Excellent [55,56,57] or almost perfect [58,59]
Gwet’s AC1	0.91	<0.01	261.7	<0.001	0.91	0.92	
Brennan and Prediger	0.85	0.01	157.9	<0.001	0.84	0.87	
Cohen/Conger’s Kappa	0.53	0.02	33.9	<0.001	0.50	0.56	Fair [56], fair to good [55], moderate [58,59], or good [57]
Krippendorff’s Alpha (preferred)	0.52	0.02	32.6	<0.001	0.49	0.55	
Scott/Fleiss’ Pi	0.52	0.02	32.4	<0.001	0.49	0.55	

## Data Availability

Individual-level raw data are unavailable due to privacy and ethical restrictions.

## Supplementary Materials

The following supporting information can be downloaded at https://www.mdpi.com/article/10.3390/ijerph21010052/s1. Table S1. The RECORD statement – checklist of items, extended from the STROBE statement, that should be reported n observational studies using routinely collected health data. Table S2. Demographic characteristics, by concordance status, QSR, 2001 to 2015, people included in regressions. Table S3. Psychiatric diagnoses and treatment, by concordance status, QSR, 2001 to 2015. Table S4. Suicide-related and life events predictors, by concordance status, QSR, 2001 to 2015. Table S5. Binary logistic regression with fractional polynomial terms for age and incident year. Table S6. Sensitivity analysis, with binary logistic regression with 5-knot restricted cubic splines for age and incident year. Table S7. Sensitivity analysis, with binary logistic regression with categorized age at death and incident year
