# Supplementary material for: Why Do We Agree to Disagree? Agreement and Reasons for Disagreement in Judgements of Intentional Self-Harm from Coroners and a Suicide Register in Queensland, Australia, from 2001 to 2015
Source: Int J Environ Res Public Health. 2023 Dec 30;21(1):52. doi: 10.3390/ijerph21010052 (PMC10815497; doi:10.3390/ijerph21010052)
Supplement: Supplementary file 1 [file ijerph-21-00052-s001.zip › ijerph-2673408-supplementary.pdf]

Supplementary Table S1. The RECORD statement – checklist of items, extended from the STROBE statement, that should be reported in observational studies using routinely collected health data

	Item No.	STROBE items	Location in manuscript where items are reported	RECORD items	Location in manuscript where items are reported
**Title and abstract**					
	1	(a) Indicate the study's design with a commonly used term in the title or the abstract<br>(b) Provide in the abstract an informative and balanced summary of what was done and what was found	Page 1 - abstract<br><br>Page 1 - abstract	RECORD 1.1: The type of data used should be specified in the title or abstract. When possible, the name of the databases used should be included.<br><br>RECORD 1.2: If applicable, the geographic region and timeframe within which the study took place should be reported in the title or abstract.<br><br>RECORD 1.3: If linkage between databases was conducted for the study, this should be clearly stated in the title or abstract.	Page 1 - abstract<br>Page 1 - title<br><br>Page 1 - abstract
**Introduction**					
Background rationale	2	Explain the scientific background and rationale for the investigation being reported	Page 1 and 2 – Introduction		
Objectives	3	State specific objectives, including any prespecified hypotheses	Page 2 – last paragraph of the introduction		
**Methods**					
Study Design	4	Present key elements of study design early in the paper	Page 2, first paragraph of 2.1. Study Design and Data Sources		
Setting	5	Describe the setting, locations, and relevant dates, including periods of recruitment, exposure, follow-up, and data collection	Page 2 and 3, first paragraph of 2.1. Study Design and Data Sources		
Participants	6	*(a) Cohort study* - Give the eligibility criteria, and the sources and methods of selection of participants. Describe methods of follow-up<br>*Case-control study* - Give the eligibility criteria, and the sources and methods of case ascertainment and control selection. Give the rationale for the choice of cases and controls<br>*Cross-sectional study* - Give the eligibility	Not applicable<br><br>Not applicable<br><br>Not applicable	RECORD 6.1: The methods of study population selection (such as codes or algorithms used to identify subjects) should be listed in detail. If this is not possible, an explanation should be provided.<br><br>RECORD 6.2: Any validation studies of the codes or algorithms used to select the population should be referenced. If validation was conducted for this study	Page 2 and 3, first and second paragraphs of 2.1. Study Design and Data Sources<br><br>Not applicable

		criteria, and the sources and methods of selection of participants<br><br>*(b) Cohort study* - For matched studies, give matching criteria and number of exposed and unexposed<br>*Case-control study* - For matched studies, give matching criteria and the number of controls per case	 <br><br><br>Not applicable<br><br><br><br><br>Not applicable	and not published elsewhere, detailed methods and results should be provided.<br><br>RECORD 6.3: If the study involved linkage of databases, consider use of a flow diagram or other graphical display to demonstrate the data linkage process, including the number of individuals with linked data at each stage.	 <br><br><br><br>Page 5, second sentence of the results. Sentence used instead of figure.
Variables	7	Clearly define all outcomes, exposures, predictors, potential confounders, and effect modifiers. Give diagnostic criteria, if applicable.	Page 3 and 4	RECORD 7.1: A complete list of codes and algorithms used to classify exposures, outcomes, confounders, and effect modifiers should be provided. If these cannot be reported, an explanation should be provided.	Codes and algorithms viewable in other online documents
Data sources/measurement	8	For each variable of interest, give sources of data and details of methods of assessment (measurement). Describe comparability of assessment methods if there is more than one group	Page 4, 2nd paragraph		
Bias	9	Describe any efforts to address potential sources of bias	Page 4, third paragraph		
Study size	10	Explain how the study size was arrived at	Page 4, 4th paragraph		
Quantitative variables	11	Explain how quantitative variables were handled in the analyses. If applicable, describe which groupings were chosen, and why	Supplementary materials (logistic regression tables) and page 4, 4th paragraph		
Statistical methods	12	(a) Describe all statistical methods, including those used to control for confounding<br><br><br><br>(b) Describe any methods used to examine subgroups and interactions<br>(c) Explain how missing data were addressed<br><br>(d) *Cohort study* - If applicable, explain how loss to follow-up was addressed<br>*Case-control study* - If applicable, explain how matching of cases and controls was addressed	not applicable for confounding Page 3, 2.2. Statistical Analysis<br><br><br><br>Not applicable<br><br><br>Page 5, results, 3rd paragraph<br><br>Not applicable<br><br><br><br>Not applicable		

		Cross-sectional study - If applicable, describe analytical methods taking account of sampling strategy<br>(e) Describe any sensitivity analyses	Not applicable<br><br>Page 4, 6th paragraph		
Data access and cleaning methods		..		RECORD 12.1: Authors should describe the extent to which the investigators had access to the database population used to create the study population.<br><br>RECORD 12.2: Authors should provide information on the data cleaning methods used in the study.	Page 4, last paragraph<br><br>Page 4, last paragraph
Linkage		..		RECORD 12.3: State whether the study included person-level, institutional-level, or other data linkage across two or more databases. The methods of linkage and methods of linkage quality evaluation should be provided.	Page 4, last paragraph
**Results**					
Participants	13	(a) Report the numbers of individuals at each stage of the study (*e.g.*, numbers potentially eligible, examined for eligibility, confirmed eligible, included in the study, completing follow-up, and analysed)<br>(b) Give reasons for non-participation at each stage.<br>(c) Consider use of a flow diagram	Page 5, first and third paragraphs of the results section	RECORD 13.1: Describe in detail the selection of the persons included in the study (*i.e.,* study population selection) including filtering based on data quality, data availability and linkage. The selection of included persons can be described in the text and/or by means of the study flow diagram.	Page 5, first and third paragraphs of the results section
Descriptive data	14	(a) Give characteristics of study participants (*e.g.*, demographic, clinical, social) and information on exposures and potential confounders<br>(b) Indicate the number of participants with missing data for each variable of interest<br>(c) *Cohort study* - summarise follow-up time (*e.g.*, average and total amount)	Supplementary Tables S2 to S4.<br><br>Not indicated<br><br>Not applicable		
Outcome data	15	*Cohort study* - Report numbers of outcome events or summary measures over time<br>*Case-control study* - Report numbers in each exposure category, or summary measures of exposure	Not applicable<br><br>Not applicable		

		Cross-sectional study - Report numbers of outcome events or summary measures	Not applicable		
Main results	16	(a) Give unadjusted estimates and, if applicable, confounder-adjusted estimates and their precision (e.g., 95% confidence interval). Make clear which confounders were adjusted for and why they were included<br>(b) Report category boundaries when continuous variables were categorized<br>(c) If relevant, consider translating estimates of relative risk into absolute risk for a meaningful time period	Not applicable<br>Not applicable<br>Supplementary Table S6<br>Relevant, but not translated		
Other analyses	17	Report other analyses done—e.g., analyses of subgroups and interactions, and sensitivity analyses	Supplementary Tables S6 and S7		
**Discussion**					
Key results	18	Summarise key results with reference to study objectives	Page 10 and 11, paragraphs 1 to 4 of the discussion.		
Limitations	19	Discuss limitations of the study, taking into account sources of potential bias or imprecision. Discuss both direction and magnitude of any potential bias	Page 11, 6th paragraph	RECORD 19.1: Discuss the implications of using data that were not created or collected to answer the specific research question(s). Include discussion of misclassification bias, unmeasured confounding, missing data, and changing eligibility over time, as they pertain to the study being reported.	Page 11, 6th paragraph
Interpretation	20	Give a cautious overall interpretation of results considering objectives, limitations, multiplicity of analyses, results from similar studies, and other relevant evidence	Page 12, paragraphs 1 and 2		
Generalisability	21	Discuss the generalisability (external validity) of the study results	Page 11, 6th paragraph		
**Other Information**					
Funding	22	Give the source of funding and the role of the funders for the present study and, if applicable, for the original study on which the present article is based	Page 12, funding statement		

Accessibility of protocol, raw data, and programming code		..		RECORD 22.1: Authors should provide information on how to access any supplemental information such as the study protocol, raw data, or programming code.	Page 12, data availability statement.

*Reference: [54] Benchimol EI, Smeeth L, Guttmann A, Harron K, Moher D, Petersen I, Sørensen HT, von Elm E, Langan SM, the RECORD Working Committee. The REporting of studies Conducted using Observational Routinely-collected health Data (RECORD) Statement. PLoS Medicine 2015; 12: e1001885.

*Checklist is protected under Creative Commons Attribution (CC BY) license

Supplementary Table S2. Demographic characteristics, by concordance status, QSR, 2001 to 2015, people included in regressions

	QSR and NCIS concordance					
	**Agreement**		**Disagreement (QSR ISH, NCIS not ISH)**		**Total**	
**Continuous variables**	**Mean**	**SD**	**Mean**	**SD**		
Age at death	43.6	17.65	44.8	16.38	na	
**Categorical variables**	**Number**	**%**	**Number**	**%**	**Number**	**%**
Sex						
Male	6580	95	365	5	6945	100
Female	1930	90	221	10	2151	100
Indigenous status						
Non-Indigenous	7879	93	564	7	8443	100
Indigenous	631	97	22	3	653	100
Marital status						
Never married	1260	94	75	6	1335	100
Married/De facto	3034	95	171	5	3205	100
Separated	1172	96	55	4	1227	100
Divorced	634	93	47	7	681	100
Widowed	295	93	22	7	317	100
Unknown	979	87	141	13	1120	100
Single	1136	94	75	6	1211	100
Employment status						
Full-time employment	1412	98	31	2	1443	100
Part-time or casual employment	428	98	11	2	439	100
Employed (unknown mode)	1108	95	57	5	1165	100
Unemployed	2044	92	167	8	2211	100
On disability pension	560	84	103	15	663	100
Retired	1082	94	72	6	1154	100
Not in labour force[1]	589	93	42	7	631	100
Unknown	1287	93	103	7	1390	100
Country of birth						
Not Australia	1667	93	127	7	1794	100
Australia	6843	94	459	6	7302	100
Remoteness area of residential address						
Major Cities of Australia	4706	93	360	7	5066	100
Inner Regional Australia	1878	93	144	7	2022	100
Outer Regional Australia	1494	95	76	5	1570	100
Remote or Very Remote Australia	432	99	6	1	438	100
Living arrangements						
With spouse	2494	95	133	5	2627	100
With friend/relative (not parents or spouse)	1334	94	86	6	1420	100
With parents	1082	94	75	6	1157	100
Other shared housing (nursing home, boarding house)	154	88	21	12	175	100
Institution (e.g. hospital or prison)	101	np	< 10	np	np	100
Alone	2167	92	180	8	2347	100
Homeless	41	79	11	21	52	100
Temporarily away from home	116	np	< 5	np	np	100
Unknown	1021	94	70	6	1091	100

np = not provided. Note that some variables required masking other values to prevent the value under 5 being derived using the row or column total. [1]Not in labor force refers to students and those working in the home.

Supplementary Table S3. Psychiatric diagnoses and treatment, by concordance status, QSR, 2001 to 2015

	QSR and NCIS concordance concerning intentional self-harm					
	**Agreement**		**Disagreement (QSR ISH, NCIS not ISH)**		**Total**	
**Categorical variables**	**Number**	**%**	**Number**	**%**	**Number**	**%**
Depression						
None known	3832	94	221	5	4053	100
Depression mentioned in case	1750	94	107	6	1857	100
Diagnosed with	2928	92	258	8	3186	100
Bipolar						
None known	8160	94	528	6	8688	100
Diagnosed	350	86	58	14	408	100
Anxiety						
None known	7954	94	512	6	8466	100
Diagnosed with	556	88	74	12	630	100
Substance use disorder						
None known	8007	94	501	6	8508	100
Diagnosed with	503	86	85	14	588	100
Neurocognitive disorder						
None known	8399	94	560	6	8959	100
Diagnosed with	111	81	26	19	137	100
GP treatment for a psychiatric condition						
Unknown	4156	94	256	6	4412	100
Yes	2424	90	255	10	2679	100
Not applicable	1930	96	75	4	2005	100
Inpatient treatment for a psychiatric condition						
Yes, current	215	95	12	5	227	100
Yes, past	1020	90	107	9	1127	100
Yes, unknown when	109	91	11	9	120	100
No/unknown	5229	93	381	7	5610	100
Not applicable	1937	96	75	4	2012	100
Outpatient treatment for a psychiatric condition						
Yes, current	941	91	88	9	1029	100
Yes, past	374	92	31	8	405	100
Yes, unknown when	195	91	19	9	214	100
No/unknown	5058	93	372	7	5430	100
Not applicable	1942	96	76	4	2018	100
Treatment from another service for a psychiatric condition						
Yes, current	671	93	50	7	721	100
Yes, past	244	94	17	6	721	100
Yes, unknown when	149	93	11	7	160	100
No/unknown	5486	93	432	7	5918	100
Not applicable	1960	96	76	4	2036	100

Supplementary Table S4. Suicide-related and life events predictors, by concordance status, QSR, 2001 to 2015

	QSR and NCIS concordance					
	Agreement		Disagreement (QSR ISH, NCIS not ISH)		Total	
**Categorical variables**	**Number**	**%**	**Number**	**%**	**Number**	**%**
Intent - lifetime						
Once or twice	2504	95	138	5	2642	100
Several times	1286	95	61	5	1347	100
No	1832	95	88	5	1920	100
Unknown	2888	91	299	9	3187	100
Intent – year						
Once or twice	2202	95	107	5	2309	100
Several times	934	97	33	3	967	100
No	2090	95	106	5	2196	100
Unknown	3284	91	340	9	3624	100
Suicide attempt - lifetime						
Once or twice	1889	94	127	6	2016	100
Several times (3 to 9)	370	92	32	8	402	100
Numerous times (10+)	36	np	< 5	np	np	100
Yes (unknown times)	161	88	21	11	182	100
Yes (multiple but unknown times)	105	np	< 20	np	np	100
No	3191	88	124	4	3315	100
Unknown	2758	91	264	9	3022	100
Suicide attempt – year						
Once or twice	1246	94	82	6	1328	100
Three or more times	168	94	11	6	179	100
Yes (unknown times)	84	np	< 10	np	np	100
Yes (multiple but unknown times)	21	np	< 5	np	np	100
Unknown	3096	91	315	9	3411	100
No	3895	96	170	4	4065	100
Alcohol consumed before suicide						
No	4534	93	341	7	4875	100
Yes	3072	94	187	6	3259	100
Unknown	904	94	58	6	962	100
Life event – relationship problems						
None known	5273	92	454	8	5727	100
Conflict	1172	96	49	4	1221	100
Separation	2065	96	83	4	2148	100
Life event – sexual abuse						
No	8348	94	580	6	8928	100
Yes	162	96	6	4	168	100
Life event – relationship conflict						
None known	7359	93	528	7	7887	100
Familial	747	95	40	5	787	100
Interpersonal	404	96	18	4	422	100
Life event – recent or pending unemployment						
No	7767	93	552	7	8319	100
Yes	743	96	34	4	777	100
Suicide note						
No	5037	90	508	9	5545	100
Yes	3172	99	45	1	3217	100
Unknown	301	90	33	10	334	100

np = not provided. Note that some variables required masking values of 5 or more to prevent the value under 5 being derived using the row total.

Supplementary Table S5. Binary logistic regression with fractional polynomial terms for age and incident year

	OR	*SE*	*Z*	*P* value	95% CI LL	95% CI UL	Sig level
Age							
Fractional polynomial term 1	1.39	0.30	1.51	0.13	0.91	2.13	
Fractional polynomial term 2	0.91	0.06	-1.54	0.12	0.80	1.03	
Incident year							
Fractional polynomial term 1	1.01	0.01	0.65	0.52	0.98	1.03	
Sex							
Male	1	.	.	.	.	.	
Female	1.94	0.20	6.45	< 0.001	1.58	2.37	***
Indigenous status							
Non-Indigenous	1	.	.	.	.	.	
Indigenous	0.61	0.15	-2.07	0.04	0.38	0.97	*
Country of birth							
Australia	1	.	.	.	.	.	
Elsewhere	1.19	0.14	1.49	0.14	0.95	1.49	
Marital status							
Married/De facto	1	.	.	.	.	.	
Never married	0.83	0.17	-0.92	0.36	0.55	1.24	
Separated	0.79	0.17	-1.10	0.27	0.51	1.21	
Divorced	0.88	0.20	-0.58	0.56	0.56	1.36	
Widowed	0.87	0.26	-0.48	0.63	0.48	1.55	
Unknown	1.44	0.27	1.97	0.05	1.00	2.07	*
Single	0.89	0.19	-0.57	0.57	0.58	1.34	
Remoteness area of residential address							
Major Cities of Australia	1	.	.	.	.	.	
Inner Regional Australia	1.19	0.13	1.53	0.13	0.95	1.48	
Outer Regional Australia	0.79	0.11	-1.71	0.09	0.60	1.04	
Remote or Very Remote Australia	0.25	0.11	-3.24	< 0.001	0.11	0.57	**
Employment status							
Unemployed	1	.	.	.	.	.	
Full-time employment	0.40	0.08	-4.33	< 0.001	0.26	0.61	***
Part-time/casual employment	0.40	0.13	-2.80	0.01	0.21	0.76	**
Employed (unknown mode)	0.74	0.13	-1.73	0.08	0.53	1.04	
On disability pension	1.85	0.29	3.99	< 0.001	1.37	2.50	***
Retired	1	0.22	0.02	0.99	0.66	1.54	
Other not in labour force	0.95	0.19	-0.25	0.80	0.64	1.41	
Unknown	0.89	0.13	-0.82	0.41	0.66	1.18	
Living arrangements							
With spouse	1	.	.	.	.	.	
With friend/relative (not parents or spouse)	1.37	0.28	1.54	0.12	0.92	2.06	
With parents	1.46	0.33	1.65	0.10	0.93	2.28	
Other shared housing (nursing home, boarding house)	2.23	0.68	2.63	0.01	1.23	4.05	**
Institution (e.g., hospital or prison)	0.97	0.48	-0.07	0.94	0.37	2.54	
Alone	1.70	0.33	2.77	0.01	1.17	2.49	**
Homeless	4.19	1.74	3.45	< 0.001	1.86	9.45	***
Temporarily away from home	.83	0.46	-0.34	0.73	0.28	2.43	
Unknown	1.03	0.22	0.15	0.88	0.68	1.56	
Depression							
No	1	.	.	.	.	.	
Depression mentioned	0.96	0.14	-0.27	0.79	0.72	1.28	
Diagnosed with	1.25	0.16	1.80	0.07	0.98	1.60	
Bipolar							

None known	1	.	.	.	.	.	
Diagnosed	1.93	.37	3.39	< 0.001	1.32	2.82	***
Anxiety							
None known	1	.	.	.	.	.	
Diagnosed with	1.32	0.2	1.80	0.07	0.98	1.79	
Substance abuse							
None known	1	.	.	.	.	.	
Yes	1.84	0.27	4.09	< 0.001	1.37	2.47	***
Dementia							
None known	1	.	.	.	.	.	
Dementia	3.67	.91	5.24	< 0.001	2.26	5.97	***
Lifetime GP treatment for a psychiatric condition							
None known	1	.	.	.	.	.	
Yes	1.41	.16	3.08	0.002	1.13	1.77	**
Not applicable	1.37	.72	0.60	0.55	0.49	3.83	
Lifetime inpatient treatment for a psychiatric condition							
None known	1	.	.	.	.	.	
Yes, current	0.59	0.20	-1.56	0.12	0.30	1.15	
Yes, past	1.01	0.14	0.06	0.95	0.77	1.33	
Yes, unknown when	1.21	0.43	0.55	0.58	0.61	2.43	
Not applicable	0.74	0.61	-0.37	0.71	0.14	3.75	
Lifetime outpatient treatment for a psychiatric condition							
None known	1	.	.	.	.	.	
Yes, current	0.92	0.13	-0.56	0.58	0.70	1.22	
Yes, past	0.99	0.22	-0.02	0.98	0.65	1.53	
Yes, unknown when	0.88	0.25	-0.45	0.65	0.51	1.52	
Not applicable	0.96	0.72	-0.05	0.96	0.22	4.20	
Other lifetime treatment for a psychiatric condition							
None known	1	.	.	.	.	.	
Yes, current	0.79	0.13	-1.39	0.17	0.56	1.10	
Yes, past	0.69	0.20	-1.28	0.20	0.40	1.22	
Yes, unknown when	0.68	0.23	-1.13	0.26	0.35	1.33	
Not applicable	1.09	0.85	0.12	0.91	0.24	4.98	
Lifetime intent							
Unknown	1	.	.	.	.	.	
Once or twice	1.01	0.18	0.07	0.94	0.71	1.44	
Several times	0.94	0.23	-0.27	0.78	0.58	1.51	
No	0.72	0.20	-1.19	0.23	0.42	1.23	
12-month intent							
Unknown	1	.	.	.	.	.	
Once or twice	0.61	0.11	-2.62	0.01	0.42	0.88	**
Several times	0.44	0.13	-2.76	0.01	0.25	0.79	**
No	1.11	0.28	0.43	0.67	0.68	1.81	
Lifetime suicide attempt							
No	1	.	.	.	.	.	
Once or twice	1.12	0.23	0.57	.57	.76	1.67	
Several times (3-9)	1.24	0.37	0.70	.48	.69	2.22	
Numerous times (10 or more)	1.15	0.78	0.20	.84	.3	4.37	
Yes (unknown times)	1.57	0.54	1.31	.19	.8	3.06	
Yes (multiple but unknown times)	1.52	0.60	1.05	.29	.7	3.3	
Unknown	1.35	.31	1.31	.19	.86	2.13	
12-month suicide attempt							
No	1	.	.	.	.	.	
Once or twice	1.14	0.24	0.63	0.53	0.76	1.71	
Three or more times	0.87	0.37	-0.32	0.75	0.38	1.99	
Yes (unknown times)	0.87	0.45	-0.28	0.78	0.32	2.37	

Yes (multiple but unknown times)	1.02	0.86	0.02	0.98	0.19	5.38	
Unknown	1.29	0.26	1.26	0.21	0.87	1.93	
Alcohol consumed before death							
No	1	.	.	.	.	.	
Yes	1.01	0.11	-0.02	0.98	0.81	1.23	
Unknown	0.85	0.13	-1.06	0.29	0.62	1.15	
Relationship problems							
None known	1	.	.	.	.	.	
Conflict	0.71	0.12	-1.96	0.05	0.50	1.00	*
Separation	0.71	0.11	-2.24	0.02	0.53	0.96	*
Experienced sexual abuse							
None known	1	.	.	.	.	.	
Yes	0.46	0.20	-1.78	0.08	0.19	1.08	
Conflict							
None known	1	.	.	.	.	.	
Familial	0.75	0.14	-1.56	0.12	0.53	1.08	
Interpersonal	0.70	0.18	-1.36	0.17	0.42	1.17	
Recent or pending unemployment							
None known	1	.	.	.	.	.	
Yes	0.72	0.14	-1.64	0.10	0.49	1.07	
Suicide note left							
No	1	.	.	.	.	.	
Yes	0.14	0.02	- 12.23	< 0.001	0.10	0.19	***
Unknown	0.96	0.19	-0.23	0.82	0.64	1.42	
Constant	0.06	0.01	-12.23	0	0.04	.1	***

Mean dependent var	0.06	SD dependent var	0.25
Pseudo r-squared	0.18	Number of obs	9096
Chi-square	792.94	Prob > chi2	0.00
Akaike crit. (AIC)	3710.41	Bayesian crit. (BIC)	4265.43

******* *p < 0.001, ** p < 0.01, * p < 0.05. Age at death term 1 = (age at death/10) ^.5-2.088809864. Age at death term 2 = (age at death/10) ^.5*ln(X)-3.077211552. Both age terms were multiplied by 10 after estimation to improve the scaling of the odds ratios. Incident year was incident year-2008.380057.*

Supplementary Table S6. Sensitivity analysis, with binary logistic regression with 5-knot restricted cubic splines for age and incident year

	OR	*SE*	*Z*	*P value*	95% CI LL	95% CI UL	Sig level
Age at death							
RCS 1	1.03	.026	1.30	0.19	0.98	1.08	
RCS 2	0.83	.162	-0.97	0.33	0.56	1.22	
RCS 3	1.59	.981	0.75	0.46	0.47	5.33	
RCS 4	0.72	.471	-0.50	0.62	0.20	2.60	
Incident year							
RCS 1	1.11	0.10	1.22	0.22	0.94	1.32	
RCS 2	1.04	0.37	0.11	0.91	0.52	2.09	
RCS 3	0.55	0.61	-0.54	0.59	0.06	4.87	
RCS 4	3.19	6.24	0.59	0.55	0.07	146.9	
Sex							
Male	1	.	.	.	.	.	
Female	1.93	.198	6.38	< 0.001	1.57	2.36	***
Indigenous status							
Non-Indigenous	1	.	.	.	.	.	
Indigenous	0.62	0.15	-1.95	0.05	0.39	1.00	
Country of birth							
Australia	1	.	.	.	.	.	
Elsewhere	1.18	0.14	1.46	0.14	0.94	1.48	
Marital status							
Married/De facto	1	.	.	.	.	.	
Never married	0.87	0.18	-0.65	0.52	0.58	1.31	
Separated	0.77	0.17	-1.18	0.24	0.50	1.19	
Divorced	0.88	0.20	-0.57	0.57	0.56	1.37	
Widowed	0.82	0.25	-0.66	0.51	0.46	1.48	
Unknown	1.37	0.26	1.67	0.09	0.95	1.97	
Single	0.83	0.18	-0.86	0.39	0.55	1.26	
Remoteness area of residential address							
Major Cities of Australia	1	.	.	.	.	.	
Inner Regional Australia	1.16	0.13	1.28	0.20	0.93	1.44	
Outer Regional Australia	0.78	0.11	-1.81	0.07	0.59	1.02	
Remote or Very Remote Australia	0.24	0.11	-3.26	0.001	0.11	0.57	**
Employment status							
Unemployed	1	.	.	.	.	.	
Full-time employment	0.43	0.09	-4.01	< 0.001	0.28	0.65	***
Part-time/casual employment	0.43	0.14	-2.56	0.01	0.23	0.82	*
Employed (unknown mode)	0.69	0.12	-2.12	.034	0.49	0.97	*
On disability pension	1.90	.295	4.15	< 0.001	1.40	2.58	***
Retired	1.01	.225	0.05	0.96	0.65	1.56	
Other not in labour force	1.00	.204	-0.01	0.99	0.67	1.49	
Unknown	.893	.132	-0.76	.445	0.66	1.19	
Living arrangements							
With spouse	1	.	.	.	.	.	
With friend/relative	1.45	0.30	1.77	0.08	0.96	2.18	
With parents	1.52	0.35	1.84	0.07	0.97	2.39	
Other shared housing (nursing home, boarding house)	2.23	0.69	2.61	0.01	1.22	4.08	**
Institution (e.g., hospital, prison)	0.98	0.48	-0.04	0.96	0.37	2.59	
Alone	1.79	0.35	2.99	0.003	1.22	2.62	**
Homeless	4.18	1.74	3.43	0.001	1.85	9.47	***
Temporarily away from home	0.89	0.49	-0.22	0.83	0.30	2.61	

Unknown	1.07	0.23	0.34	0.74	0.71	1.63	
Depression							
No	1	.	.	.	.	.	
Depression mentioned	0.94	0.14	-0.40	0.69	0.71	1.26	
Diagnosed with	1.18	0.15	1.32	0.19	0.9	1.51	
Bipolar							
None known	1	.	.	.	.	.	
Diagnosed with	1.88	0.36	3.23	0.001	1.28	2.75	**
Anxiety							
None known	1	.	.	.	.	.	
Diagnosed with	1.36	0.21	1.99	0.05	1.01	1.85	*
Substance abuse							
None known	1	.	.	.	.	.	
Yes	1.83	0.27	4.04	< 0.001	1.37	2.46	***
Dementia							
None known	1	.	.	.	.	.	
Diagnosed with	3.75	0.94	5.28	< 0.001	2.298	6.134	***
Lifetime GP treatment for a psychiatric condition							
None known	1	.	.	.	.	.	
Yes	1.41	0.16	3.03	0.002	1.13	1.76	**
Not applicable	1.35	0.71	0.57	0.57	0.48	3.81	
Lifetime inpatient treatment							
None known	1	.	.	.	.	.	
Yes, current	0.63	0.22	-1.35	0.18	0.32	1.23	
Yes, past	1.00	0.14	-0.01	1.00	0.76	1.32	
Yes, unknown when	1.39	0.50	0.93	0.35	0.69	2.80	
Not applicable	0.78	0.64	-0.30	0.76	0.15	3.95	
Lifetime outpatient treatment for a psychiatric condition							
None known	1	.	.	.	.	.	
Yes, current	0.86	0.12	-1.04	0.30	0.65	1.14	
Yes, past	0.98	0.21	-0.10	0.92	0.64	1.50	
Yes, unknown when	0.92	0.26	-0.29	0.77	0.53	1.59	
Other lifetime treatment for a psychiatric condition							
None known	1	.	.	.	.	.	
Not applicable	0.93	0.70	-0.10	0.92	0.02	4.07	
Yes, current	0.80	0.14	-1.32	0.19	0.57	1.12	
Yes, past	0.73	0.21	-1.11	0.27	0.42	1.28	
Yes, unknown when	0.75	0.25	-0.84	0.40	0.38	1.46	
Not applicable	0.99	0.77	-0.01	0.99	0.22	4.50	
Lifetime intent							
Unknown	1	.	.	.	.	.	
Once or twice	1.01	0.18	0.07	0.95	0.71	1.44	
Several times	0.89	0.22	-0.46	0.65	0.55	1.45	
No	0.73	0.20	-1.13	0.26	0.42	1.26	
12-month intent							
Unknown	1	.	.	.	.	.	
Once or twice	0.63	0.12	-2.48	0.01	0.43	0.91	*
Several times	0.48	0.14	-2.47	0.01	0.27	0.86	*
No	1.16	0.29	0.59	0.56	0.71	1.91	
Lifetime suicide attempt							
No	1	.	.	.	.	.	
Once or twice	1.14	0.23	0.65	0.52	0.77	1.69	
Several times (3-9)	1.24	0.37	0.72	0.47	0.69	2.24	
Numerous times (10 or more)	1.16	0.80	0.22	0.83	0.30	4.48	
Yes (unknown times)	1.60	0.55	1.37	0.17	0.82	3.13	
Yes (multiple but unknown times)	1.43	0.57	0.89	0.37	0.65	3.12	
Unknown	1.40	0.33	1.42	0.16	0.88	2.21	

12-month suicide attempt							
No	1	.	.	.	.	.	
Once or twice	1.15	0.24	0.66	0.51	0.76	1.72	
Three or more times	0.89	0.37	-0.29	0.77	0.39	2.02	
Yes (unknown times)	0.93	0.48	-0.15	0.88	0.34	2.55	
Yes (multiple but unknown times)	1.17	0.99	0.18	0.86	0.22	6.11	
Unknown	1.26	0.26	1.12	0.26	0.84	1.89	
Alcohol consumed before death							
No	1	.	.	.	.	.	
Yes	1.00	0.10	0.04	0.97	0.83	1.24	
Unknown	0.88	0.14	-0.77	0.04	0.64	1.21	
Relationship problems							
None known	1	.	.	.	.	.	
Conflict	0.72	0.13	-1.86	0.06	0.51	1.02	
Separation	0.72	0.11	-2.22	0.03	0.53	0.96	*
Experienced sexual abuse							
None known	1	.	.	.	.	.	
Yes	0.46	0.20	-1.76	0.08	0.19	1.09	
Conflict							
None known	1	.	.	.	.	.	
Familial	0.76	0.14	-1.48	0.14	0.53	1.09	
Interpersonal	0.72	0.19	-1.27	0.20	0.43	1.20	
Recent or pending unemployment							
None known	1	.	.	.	.	.	
Yes	0.76	0.15	-1.35	0.18	0.52	1.13	
Suicide note left							
No	1	.	.	.	.	.	
Yes	0.14	0.02	-12.17	< 0.001	0.10	0.19	***
Unknown	1.03	0.21	0.12	0.90	0.69	1.53	
Constant	0	0	-1.25	0.21	0	6.888e+53	

Mean dependent var	0.064	SD dependent var	0.246
Pseudo r-squared	0.187	Number of obs	9096
Chi-square	813.028	Prob > chi2	0.000
Akaike crit. (AIC)	3700.322	Bayesian crit. (BIC)	4290.916

**** p < 0.001, ** p < 0.01, * p < 0.05. RCS: Restricted cubic spline with 5 knots.*

Supplementary Table S7. Sensitivity analysis, with binary logistic regression with categorized age at death and incident year

	OR	*SE*	*Z*	*P value*	95% CI LL	95% CI UL	Sig level
10-year age group							
5-14	1.00	.	.	.	.	.	
15-24	0.51	0.30	-1.16	0.25	0.17	1.58	
25-34	0.70	0.41	-0.62	0.53	0.22	2.18	
35-44	0.64	0.38	-0.75	0.45	0.20	2.03	
45-54	0.65	0.39	-0.72	0.47	0.21	2.09	
55-64	0.62	0.37	-0.79	0.43	0.19	2.02	
65-74	0.53	0.33	-1.04	0.30	0.16	1.78	
75 and over	0.50	0.31	-1.10	0.27	0.14	1.72	
Incident year							
2001 to 2005	1	.	.	.	.	.	
2006 to 2010	1.31	0.17	2.08	0.04	1.02	1.68	*
2011 to 2015	0.98	0.13	-0.16	0.87	0.75	1.27	
Sex							
Male	1	.	.	.	.	.	
Female	1.95	0.20	6.51	< 0.001	1.59	2.38	***
Indigenous status							
Non-Indigenous	1	.	.	.	.	.	
Indigenous	0.61	0.15	-2.02	0.04	0.38	0.98	*
Country of birth							
Australia	1	.	.	.	.	.	
Elsewhere	1.18	0.14	1.44	0.15	0.94	1.48	
Marital status							
Married/De facto	1	.	.	.	.	.	
Never married	0.84	0.17	-0.83	0.41	0.56	1.26	
Separated	0.80	0.18	-1.03	0.31	0.52	1.23	
Divorced	0.89	0.20	-0.49	0.62	0.57	1.40	
Widowed	0.85	0.25	-0.57	0.57	0.47	1.51	
Unknown	1.45	0.27	1.98	0.05	1.00	2.08	*
Single	0.90	0.19	-0.51	0.61	0.59	1.36	
Remoteness area of residential address							
Major Cities of Australia	1	.	.	.	.	.	
Inner Regional Australia	1.17	0.13	1.37	0.17	0.94	1.46	
Outer Regional Australia	0.77	0.11	-1.83	0.07	0.59	1.02	
Remote or Very Remote Australia	0.24	0.11	-3.27	< 0.001	0.10	0.57	**
Employment status							
Unemployed	1	.	.	.	.	.	
Full-time employment	0.42	0.09	-4.12	< 0.001	0.27	0.63	***
Part-time/casual employment	0.41	0.14	-2.69	0.01	0.22	0.79	**
Employed (unknown mode)	0.70	0.12	-2.03	0.04	0.50	0.99	*
On disability pension	1.85	0.29	3.98	< 0.001	1.37	2.51	***
Retired	1.01	0.22	0.03	0.98	0.65	1.56	
Other not in labour force	0.90	0.18	-0.52	0.61	0.60	1.35	
Unknown	0.89	0.13	-0.79	0.43	0.67	1.19	
Living arrangements							
With spouse	1	.	.	.	.	.	
With friend/relative	1.39	.29	1.59	0.11	0.93	2.09	
With parents	1.44	.33	1.58	0.11	0.92	2.25	
Other shared housing (nursing home, boarding house)	2.16	.66	2.52	0.01	1.19	3.94	*
Institution (e.g., hospital, prison)	1.00	0.49	-0.00	1.00	0.38	2.64	
Alone	1.70	0.33	2.75	0.01	1.17	2.49	**
Homeless	4.17	1.74	3.42	< 0.001	1.84	9.44	***
Temporarily away from home	0.85	0.47	-0.29	0.77	0.29	2.50	
Unknown	1.03	0.22	0.12	0.90	0.68	1.55	
Depression							
No	1	.	.	.	.	.	
Depression mentioned	0.96	0.14	-0.26	0.79	0.72	1.28	
Diagnosed with	1.23	0.16	1.66	0.10	0.96	1.58	
Bipolar							
None known	1	.	.	.	.	.	

Diagnosed with	1.94	0.38	3.40	< 0.001	1.32	2.84	***
Anxiety							
None known	1	.	.	.	.	.	
Diagnosed with	1.37	0.21	2.03	0.04	1.01	1.86	*
Substance abuse							
None known	1	.	.	.	.	.	
Yes	1.86	0.28	4.15	< 0.001	1.39	2.49	***
Dementia							
None known	1	.	.	.	.	.	
Dementia	3.72	0.92	5.30	< 0.001	2.29	6.06	***
Lifetime GP treatment for a psychiatric condition							
None known	1	.	.	.	.	.	
Yes	1.43	0.16	3.16	< 0.001	1.15	1.78	**
Not applicable	1.38	0.74	0.61	0.54	0.49	3.94	
Lifetime inpatient treatment for a psychiatric condition							
None known	1	.	.	.	.	.	
Yes, current	0.61	0.21	-1.45	0.15	0.31	1.19	
Yes, past	1.02	0.14	0.12	0.91	0.77	1.34	
Yes, unknown when	1.28	0.45	0.68	0.49	0.64	2.56	
Not applicable	0.79	0.66	-0.28	0.78	0.15	4.09	
Lifetime outpatient treatment for a psychiatric condition							
None known	1	.	.	.	.	.	
Yes, current	0.9	0.13	-0.71	0.48	0.68	1.20	
Yes, past	1.00	0.22	0.01	0.99	0.65	1.54	
Yes, unknown when	0.91	0.25	-0.34	0.73	0.53	1.57	
Other lifetime treatment for a psychiatric condition							
None known	1	.	.	.	.	.	
Not applicable	0.92	0.70	-0.11	0.91	0.21	4.12	
Yes, current	0.79	0.13	-1.39	0.17	0.56	1.10	
Yes, past	0.72	0.21	-1.14	0.25	0.41	1.26	
Not applicable	1.04	0.81	0.05	0.96	0.22	4.80	
Yes, unknown when	0.70	0.24	-1.07	0.29	0.36	1.35	
Lifetime intent							
Unknown	1	.	.	.	.	.	
Once or twice	1.03	0.19	0.16	0.87	0.72	1.47	
Several times	0.94	0.23	-0.23	0.82	0.58	1.53	
No	0.75	0.21	-1.03	0.30	0.44	1.29	
12-month intent							
Unknown	1	.	.	.	.	.	
Once or twice	0.61	0.11	-2.64	0.01	0.42	0.88	**
Several times	0.44	0.13	-2.75	0.01	0.25	0.79	**
No	1.13	0.28	0.48	0.63	0.69	1.84	
Lifetime suicide attempt							
No	1	.	.	.	.	.	
Once or twice	1.15	0.23	0.70	0.48	0.78	1.71	
Several times (3-9)	1.28	0.39	0.84	0.40	0.71	2.31	
Numerous times (10 or more)	1.18	0.81	0.24	0.81	0.31	4.53	
Yes (unknown times)	1.58	0.54	1.33	0.18	0.81	3.08	
Yes (multiple unknown times)	1.49	0.59	1.00	0.32	0.68	3.25	
Unknown	1.43	.33	1.53	0.13	0.90	2.25	
12-month suicide attempt							
No	1	.	.	.	.	.	
Once or twice	1.14	0.24	0.65	0.52	0.76	1.72	
Three or more times	0.87	0.36	-0.34	0.73	0.38	1.97	
Yes (unknown times)	0.90	0.46	-0.21	0.84	0.33	2.47	
Yes (multiple unknown times)	1.03	0.87	0.04	0.97	0.20	5.41	
Unknown	1.27	0.26	1.15	0.25	0.85	1.89	
Alcohol consumed before death							
No	1	.	.	.	.	.	
Yes	1.01	0.11	0.06	0.95	0.82	1.24	
Unknown	0.88	0.14	-0.80	0.42	0.64	1.20	
Relationship problems							
None known	1	.	.	.	.	.	
Conflict	0.73	0.13	-1.79	0.07	0.52	1.03	
Separation	0.73	0.11	-2.08	0.04	0.54	0.98	*
Experienced sexual abuse							

None known	1	.	.	.	.	.	
Yes	0.46	0.20	-1.74	0.08	0.19	1.10	
Conflict							
None known	1	.	.	.	.	.	
Familial	0.75	0.14	-1.55	0.12	0.53	1.08	
Interpersonal	0.71	0.19	-1.30	0.19	0.43	1.19	
Recent or pending unemployment							
None known	1	.	.	.	.	.	
Yes	0.75	0.15	-1.45	0.15	0.51	1.11	
Suicide note left							
No	1	.	.	.	.	.	
Yes	0.14	0.02	-12.23	< 0.001	0.10	0.19	***
Unknown	0.97	0.20	-0.16	0.88	0.65	1.44	
Constant	0.08	0.05	-3.99	< 0.001	0.02	0.28	***

Mean dependent var	0.06	SD dependent var	0.25
Pseudo r-squared	0.18	Number of obs	9096
Chi-square	802.84	Prob > chi2	0.00
Akaike crit. (AIC)	3712.51	Bayesian crit. (BIC)	4310.22

*** *p < 0.001*, ** *p < 0.01*, * *p < 0.05*
